# Africanized Honey Bee Sting-Induced Stress-Related Cardiomyopathy: A Bee or Octopus Trap

**DOI:** 10.7759/cureus.16681

**Published:** 2021-07-28

**Authors:** Rajeev V Seecheran, Ryan Ramdin, Sonia Singh, Valmiki Seecheran, Sangeeta Persad, Lakshmipathi Peram, Sadi S Raza, Naveen A Seecheran

**Affiliations:** 1 Internal Medicine, Eric Williams Medical Sciences Complex, Champs Fleurs, TTO; 2 Cardiology, HeartPlace Dallas, Dallas, USA; 3 Cardiology, Eric Williams Medical Sciences Complex, Champs Fleurs, TTO

**Keywords:** africanized honey bee, envenomation, hymenoptera, stress-related cardiomyopathy, stress-induced cardiomyopathy, takotsubo's syndrome

## Abstract

The Africanized honey bee commonly referred to as the "killer bee," is a hybrid of the East African lowland honey bee with various European honey bees. These bees tend to be more hostile as compared to other bee species. Their stings may have devastating clinical sequelae, including cardiovascular, cerebrovascular events, and fatalities. We report the first-in-Caribbean case of a middle-aged woman who experienced stress-related, Takotsubo cardiomyopathy (also known as stress-related cardiomyopathy [SRC]) after being afflicted with innumerable Africanized honey bee stings.

Key clinical message: The clinician should be cognizant of Takotsubo's cardiomyopathy as a potential sequela of Hymenoptera envenomation and anaphylaxis.

## Introduction

The Africanized honey bee is rapidly looming as an environmental health care issue in the Caribbean [1]. These crossbred honey bees are generally hostile and intrusive, often inflicting a more painful sting and higher risk of envenomation [2].

These bee swarm invasions can precipitate cardiovascular and cerebrovascular events, even culminating in death [1]. The literature is not replete describing anaphylaxis-associated, stress-induced Takotsubo syndrome with only a paucity of case reports [3-5]. Herein, we report the first-in-Caribbean case of a middle-aged woman who experienced stress-related cardiomyopathy (SRC) after being afflicted with innumerable Africanized honey bee stings.

## Case presentation

**Figure 1:**
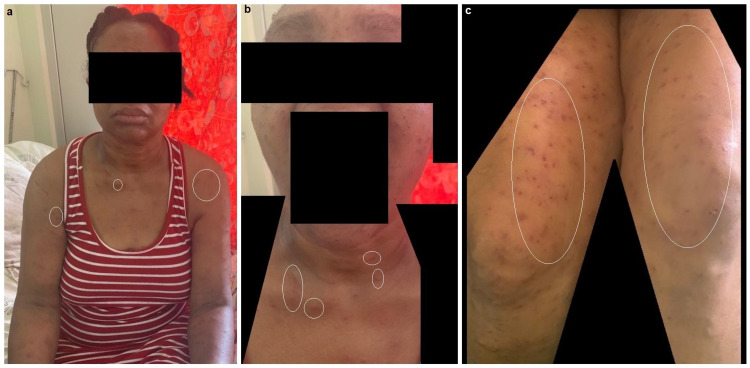
Patient's stings throughout her body during her recovery. (a) Frontal view aspect of the patient's stings involving the entirety of her body (some lesions encircled in white). (b) Close-up view of patient's face and upper neck displaying healing lesions with mild induration and erythema. (c) Multiple bee stings on upper thigh regions through various stages of healing.

A 48-year-old Caribbean-Black female with no significant medical history presented to the emergency department via family members en extremis, obtunded and somnolent. Her vital signs indicated systolic blood pressures of 98 mm Hg, heart rate of 105 beats/min, respiratory rate of 26 breaths/min, and oxygen saturation of 94% on supplemental oxygen. Her physical examination revealed hundreds of stings throughout her body, including her face, upper chest, and lower extremities (see Figures 1a-1c). The emergency medicine team reported that the patient was attacked by a swarm of Africanized honey bees at home, circa 3-4 hours prior, and did not receive epinephrine en route.

**Figure 2:**
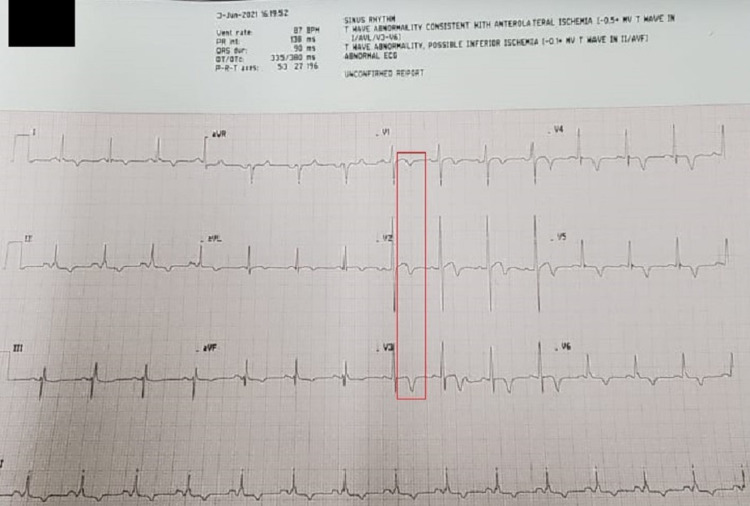
Patient's 12-lead electrocardiogram. The red box illustrates the subtle 1-2 mm ST-segment elevation with T-wave inversions in the precordial leads, a common electrocardiographic finding in Takotsubo's cardiomyopathy.

**Figure 3:**
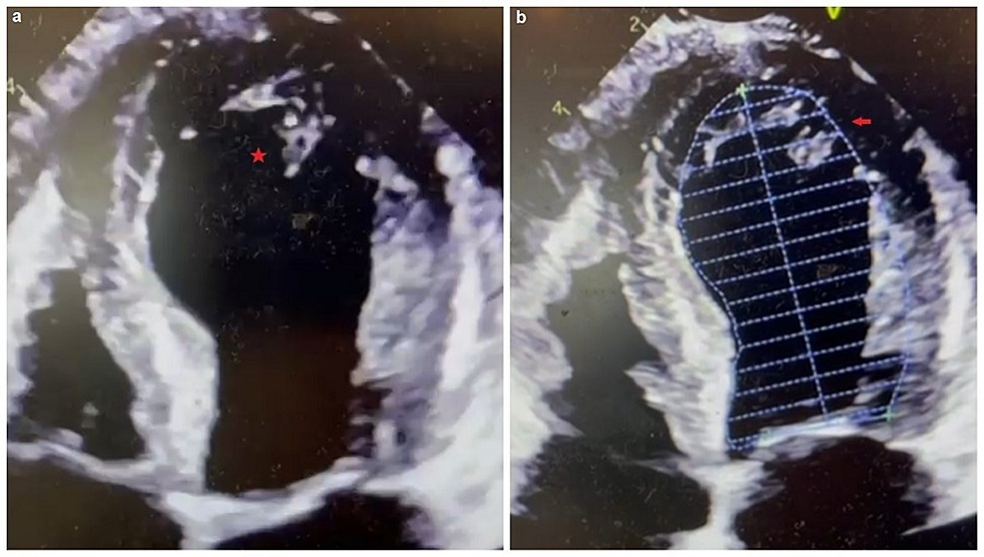
Patient's transthoracic echocardiogram. (a) Apical four-chamber view demonstrating the severe apical akinesis with left ventricular dilatation as depicted by the red star (apical ballooning). (b) Apical four-chamber view with endocardial tracing outline illustrating the similarity to the Japanese octopus trap as depicted by the red arrow.

A 12-lead electrocardiogram (ECG) revealed sinus rhythm with subtle 1-2 mm ST-segment myocardial elevation in V1-V3 with diffuse T-wave inversions (Figure 2). A portable chest radiograph indicated mild interstitial edema without cardiomegaly or pleural effusions. Pertinent diagnostic laboratory investigations included a d-dimer 2,583 ng/dL (normal ≤ 500 ng/mL), N-terminal-pro-brain natriuretic peptide 3,674 pg/mL (normal ≤ 300 pg/mL), troponin I 1.49 ng/mL (normal < 0.15 ng/mL). Her complete blood count revealed a mild leukocytosis with normal hemoglobin and platelet count. Renal function tests, including random blood glucose and hepatic function panel, were relatively normal. A bedside two-dimensional transthoracic echocardiogram (2D-TTE) demonstrated severe apical "ballooning" with an estimated ejection fraction of 20%-25% (Figures 3a, 3b). SARS-CoV-2 IgM and IgM antibody serologies (Abbott Laboratories, Chicago, IL, USA) were negative.

**Figure 4:**
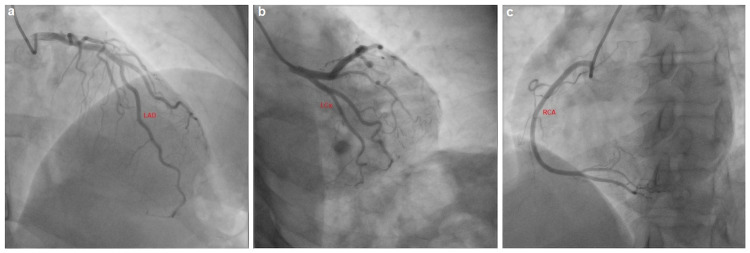
Patient's coronary angiogram. (a) Angiographically normal left anterior descending (LAD) coronary artery. (b) Angiographically normal left circumflex (LCx) coronary artery. (c) Angiographically normal right coronary artery (RCA).

The patient was subsequently admitted to the cardiac care unit and initiated on a norepinephrine infusion, where her mental state improved to being oriented and fully conversant. She was immediately administered intravenous antihistamines (diphenhydramine 50 mg every six hours) and glucocorticoids (hydrocortisone 200 mg every eight hours). Her blood pressures stabilized hemodynamically within the ensuing hour, and she then proceeded to urgent coronary angiography, which revealed angiographically normal coronary arteries (Figures 4a-4c). Additionally, she was transitioned to comprehensive, guideline-directed medical therapy for the tentative diagnosis of SRC and Takotsubo's syndrome as she gradually became normotensive. This included low-dose valsartan/sacubitril 50 mg, eplerenone 25 mg, bisoprolol 2.5 mg, and dapagliflozin 5 mg. Her antihistaminergic and steroid therapies were subsequently weaned to an oral regimen during her inpatient one-week hospitalization. She was eventually discharged with the aforementioned neurohormonal inhibition. On her scheduled follow-up appointment two weeks later, her interval surveillance echocardiogram revealed normalization of the left ventricular ejection fraction to 60%.

## Discussion

Africanized honey bees belong to the Apoidea winged Hymenoptera family [6]. Envenomation may ensue in a “cytokine storm,” resulting in multisystemic inflammatory syndrome. Typically, anaphylaxis has a prevalence of less than 5% and usually manifests in excess of 50-100 stings [7]. Adolescence ( age < 20 years) and male gender are negatively associated with increased mortality [8]. Our patient's clinical trajectory was consistent with Takotsubo's syndrome, aptly named due to the left ventricle's morphologic similarity to a Japanese octopus trap [9,10]. Kounis syndrome, which has been implicated in SRC, was also considered a differential diagnosis; however, no coronary vasospasm was apparent on coronary angiography [11].

The patient's pathophysiology may have resulted from either a direct envenomation effect, anaphylaxis, excessive exogenous or endogenous epinephrine. SRC has been observed with hymenopterous-induced anaphylaxis, but central to these reports has been the administration of exogenous epinephrine (0.2-5 mg) during the emergent resuscitation [12-14]. It is worthwhile to note that various phenotypes of SRC do clinically manifest, including "reverse Takotsubo syndrome" with mid-basal akinesis [14]. Our patient's classical left ventricular apical ballooning was echocardiographically diagnosed before she was initiated on the norepinephrine infusion, thus refuting the rationale for excessive exogenous epinephrine. We postulate that the patient experienced more than 200 stings which stimulated an endogenous catecholamine storm with an estimated envenomation toxin volume of 60 mg (0.3 mg per sting) [8]. Murine studies revealed a marked decrease in tissue noradrenaline concentration when injected with Africanized honey bee venom, suggesting its intracellular release [5]. It is also biologically plausible that the SRC may have resulted from a direct toxic effect of the bee venom, which may precipitate vascular thromboembolic phenomenon, refractory hypotension, malignant hypertension, arrhythmias, and hypercoagulability. Its constituents comprise histamine, serotonin, thromboxanes, leukotrienes, polypeptide toxins, hyaluronidase, phospholipase enzymes which can induce vasoconstriction and disrupt cell integrity [1,15].

The patient was treated with supportive measures, including norepinephrine, histamine receptor blockade, and parenteral glucocorticoids before being tapered and transitioned to an oral regimen. However, there are no specific evidence-based guidelines to define an optimal medical regimen for SRC [16]. Preemptively, the patient is treated with standard heart failure therapies until there is a recovery of systolic function, which usually occurs in 1-4 weeks. As such, our patient was treated with the "fantastic four" combination of an angiotensin-receptor, neprilysin inhibitor, beta-blocker, mineralocorticoid receptor antagonist, and sodium-glucose-like type cotransporter inhibitor to derive cardiovascular benefit with respect to mortality and morbidity, including heart failure hospitalizations [17].

## Conclusions

We report the first-in-Caribbean case of a middle-aged woman who experienced stress-related, Takotsubo cardiomyopathy after being afflicted with a cluster of Africanized honey bee stings which was successfully managed with emergency resuscitative and supportive measures followed by the gradual introduction of neurohormonal therapies. The clinician should be cognizant of Takotsubo's cardiomyopathy as a potential sequela of Hymenoptera envenomation and anaphylaxis.

## References

[1] Ramlackhansingh AF, Seecheran N (2020). Africanised honey bee sting-induced ischaemic stroke. BMJ Case Rep.

[2] Ferreira RS Jr, Almeida RAMB, Barraviera SRCS, Barraviera B (2012). Historical perspective and human consequences of Africanized bee stings in the Americas. J Toxicol Environ Health B Crit Rev.

[3] Aono J, Saito M, Inaba S (2019). Multiple bee sting-induced life-threatening Takotsubo cardiomyopathy. Circ J.

[4] Mishra S, Mishra A, Mishra JP (2016). Bee sting: a rare cause of Takotsubo cardiomyopathy. Int J Cardiol.

[5] Ghanim D, Adler Z, Qarawani D (2015). Takotsubo cardiomyopathy caused by epinephrine-treated bee sting anaphylaxis: a case report. J Med Case Rep.

[6] Fan HW, Kalil J (2016). Massive bee envenomation. Critical Care Toxicology.

[7] Bourgain C, Pauti MD, Fillastre JP (1998). Massive poisoning by African bee stings. Presse Med.

[8] Kalyoncu AF, Fuat Kalyoncu A, Uğur Demir A (1997). Bee and wasp venom allergy in Turkey. Ann Allergy Asthma Immunol.

[9] Akashi YJ, Nef HM, Möllmann H (2010). Stress cardiomyopathy. Annu Rev Med.

[10] Tsuchihashi K, Ueshima K, Uchida T (2001). Transient left ventricular apical ballooning without coronary artery stenosis: a novel heart syndrome mimicking acute myocardial infarction. J Am Coll Cardiol.

[11] Yanagawa Y, Nishi K, Tomiharu N (2009). A case of takotsubo cardiomyopathy associated with Kounis syndrome. Int J Cardiol.

[12] Manivannan V, Li JTC, Prasad A (2009). Apical ballooning syndrome after administration of intravenous epinephrine during an anaphylactic reaction. Mayo Clinic Proc.

[13] Han Y, Yeon S (2006). Midventricular hypokinesis as a cardiac manifestation of anaphylaxis: a case report. J Am Soc Echocardiogr.

[14] Abraham J, Mudd JO, Kapur NK (2009). Stress cardiomyopathy after intravenous administration of catecholamines and beta-receptor agonists. J Am Coll Cardiol.

[15] Crawley F, Schon F, Brown MM (1999). Cerebral infarction: a rare complication of wasp sting. J Neurol Neurosurg.

[16] Bybee KA, Kara T, Prasad A (2004). Systematic review: transient left ventricular apical ballooning: a syndrome that mimics ST-segment elevation myocardial infarction. Ann Intern Med.

[17] Bauersachs J (2021). Heart failure drug treatment: the fantastic four. Eur Heart J.

